# Commentary by the Commission for Hospital Hygiene and Infection Prevention (KRINKO) on the indication-based use of disposable medical gloves in the healthcare sector

**DOI:** 10.3205/dgkh000510

**Published:** 2024-11-05

**Authors:** Commission for Hospital Hygiene and Infection Prevention (KRINKO)

**Affiliations:** 1 Robert Koch Institute, Berlin, Germany

**Keywords:** disposable medical gloves, sustainability, indications, misindications, glove disinfection, glove selection, hand hygiene

## Abstract

**Introduction:**

When using disposable medical gloves, the indications for their use are not always clear in practice, so that they are often worn even in cases where this is neither necessary for the protection of the patient nor for self-protection. This can not only lead to neglect of adequate hand hygiene, but is also ecologically critical in terms of glove consumption and the resources used.

**Method:**

German and international recommendations, including WHO guidelines and information, statements and separate publications on indications and non-indications of disposable medical gloves were evaluated as the basis for deriving the indications for wearing disposable medical gloves.

**Results:**

Typical indications for disposable medical gloves for direct and indirect patient contact, laboratory work and other medical activities are summarized in a table. Situations in which the use of disposable medical gloves is not indicated are also shown separately in tabular form.

**Discussion:**

Further situations are discussed in which the wearing of disposable medical gloves is currently recommended from an infection prevention or occupational health and safety perspective, but should be re-evaluated in the future.

In addition to other aspects for reducing glove consumption, such as glove disinfection, guidelines for implementing the use of gloves according to indication are also presented, emphasizing the special role-model function of hygiene staff.

**Conclusion:**

By avoiding the use of disposable medical gloves where there is no indication and by selecting suitable glove material where there is an indication, not only can sustainability be increased, but costs can also be saved without jeopardizing patient and occupational safety.

## Zusammenfassung

**Einleitung:**

Beim Einsatz medizinischer Einmalhandschuhe sind die Indikationen für ihren Einsatz *in praxi* nicht immer klar, so dass sie häufig getragen werden, obwohl das weder für den Schutz des Patienten noch für den Selbstschutz erforderlich ist. Das kann nicht nur zur Vernachlässigung adäquater Händehygiene führen, sondern ist in Hinblick auf den Handschuhverbrauch und die damit aufgewendeten Ressourcen auch ökologisch kritisch zu betrachten.

**Methode:**

Als Grundlage für die Ableitung der Indikationen zum Tragen von medizinischen Einmalhandschuhen wurden deutsche und internationale Empfehlungen einschließlich Richtlinien und Informationen der WHO, Stellungnahmen sowie Einzelveröffentlichungen zu Indikationen und Nichtindikationen von medizinischen Einmalhandschuhen ausgewertet.

**Ergebnisse:**

Tabellarisch werden typische Indikationen für medizinische Einmalhandschuhe bei direktem und indirektem Patientenkontakt, bei Labortätigkeit und bei anderen medizinischen Tätigkeiten zusammengefasst. Separat werden ebenfalls tabellarisch Situationen dargestellt, in denen das Tragen medizinischer Einmalhandschuhe nicht indiziert ist.

**Diskussion:**

Weiterführend werden Situationen diskutiert, in denen das Tragen medizinischer Einmalhandschuhe aus infektionspräventiver bzw. arbeitsschutzrechtlicher Sicht derzeit empfohlen wird, aber zukünftig reevaluiert werden sollte.

Neben weiteren Aspekten zur Reduktion des Handschuhverbrauchs wie der Handschuhdesinfektion werden auch Orientierungshilfen für die Implementierung für einen indikationsgerechten Handschuheinsatz unter Hervorhebung der besonderen Vorbildfunktion der Hygienebeauftragten aufgezeigt.

**Schlussfolgerung:**

Durch den Verzicht auf medizinische Einmalhandschuhe bei fehlender Indikation und die Auswahl geeigneten Handschuhmaterials bei gegebener Indikation können nicht nur die Nachhaltigkeit gesteigert, sondern zugleich Kosten gespart werden, ohne dass die Patienten- und Arbeitssicherheit gefährdet wird.

## Table of contents

1 Background and objectives

2 Introduction

3 Definition: Disposable medical gloves and distinction from other gloves

4 Indications and misindications for the use of disposable medical gloves

4.1 Situations in which the wearing of disposable medical gloves is currently recommended from an infection prevention or occupational health and safety perspective, but could be re-evaluated in the future

5 Further aspects: Disinfection of disposable medical gloves and material selection

6 Implementation and options for improving the implementation of the indication-based wearing of disposable medical gloves

References

### Legal notice

This translation is intended solely to provide information to the interested, non-German-reading public. Any discrepancies or differences that may arise in translation of the official German version “Kommentar der Kommission für Krankenhaushygiene und Infektionsprävention (KRINKO) zum indikationsgerechten Einsatz medizinischer Einmalhandschuhe im Gesundheitswesen“ (Epid Bull 2024;10:3-15; DOI: 10.25646/11984) are not binding and have no legal effect.

### Legal notice in German

**Rechtlicher Hinweis**

Rechtlich bindend ist die deutsche Originalfassung “Kommentar der Kommission für Krankenhaushygiene und Infektionsprävention (KRINKO) zum indikationsgerechten Einsatz medizinischer Einmalhandschuhe im Gesundheitswesen“ (Epid Bull 2024;10:3-15; DOI: 10.25646/11984). Die englische Fassung dient der Information der internationalen Fachöffentlichkeit.

## 1 Background and objectives

The use of disposable medical gloves in the healthcare sector is addressed in various recommendations of the Commission for Hospital Hygiene and Infection Prevention (KRINKO), for example, in the recommendations “Infection prevention in the care and treatment of patients with communicable diseases”, “Hand hygiene in healthcare facilities” and “Hygiene requirements for punctures and injections” as well as in the associated commentary [1], [2], [3], [4]. When implementing the recommendations, it is often observed that the indications for the use of disposable medical gloves are not always clear. In practice, this results in situations in which disposable medical gloves are worn even in cases where this is not necessary for patient protection and/or self-protection, i.e., there is no indication. This should be viewed critically, not only from an ecological perspective but also from an infection prevention perspective. On the one hand, excessive, unreflected glove wearing can result in infection risks due to the neglect of adequate hand hygiene, for example, if gloves are not changed and hand antisepsis is not carried out as indicated. On the other hand, these incorrect indications, which lead to excessive glove use, have a direct negative impact on the climate, the environment and employees.

The KRINKO has therefore decided to provide a commentary on the indications for wearing disposable medical gloves with the aim of raising awareness of situations in which gloves should be worn or in which wearing them is not absolutely necessary from an infection prevention perspective. Ideally, this commentary will help users on site to identify co-benefit strategies, i.e. measures that are preferable from the point of view of patient and occupational safety as well as climate and environmental protection.

## 2 Introduction

The healthcare sector in Germany is responsible for 5.2% of the country's climate-damaging emissions [5]. Worldwide, the healthcare sector contributes 4.4% to global greenhouse gas emissions. If the healthcare sector were an independent nation, it would rank fifth among the world’s greatest greenhouse-gas emitters [5]. The current status report of the Robert Koch Institute (RKI) “Climate Change and Health 2023” impressively illustrates the extent to which human health, particularly regarding infection prevention, is directly and indirectly influenced by the consequences of climate change [6], [7], [8], [9], [10], [11].

The World Health Organization (WHO) perceives a great potential to counteract climate change through the sensible use of resources in the healthcare sector [12]. Apart from the social goal of climate and environmental protection, medical and social institutions have a moral and ethical obligation to assume ecological responsibility due to their competence. In this context, administrative, clinical and organizational processes as well as infrastructures should be examined for opportunities to promote health, climate and environmental protection through a contemporary interpretation of sustainability strategies. Protection against infection is also an implicit sustainability protection goal of medical facilities. **Protecting patients from infections and infectious diseases is the primary protection goal of the KRINKO. **

The reduction of healthcare-associated infections (HAI) and unwanted nosocomial colonization is the core task of hygiene and therefore a central task in the healthcare system. This requires various measures and interventions, which in turn that need the corresponding resources. Nosocomial infections (NI) represent an enormous challenge for all patients, relatives, healthcare professionals, and society at large [13]. A recent study put the number of new cases of HAI across Europe at circa 2.5 million per year [14]. For Germany, the annual number of HAI is estimated at around 400,000–600,000 and the resulting deaths at circa 10,000–20,000 [15]. Data from the German point-prevalence survey on HAI from 2022 revealed a prevalence of 3.6% of patients with HAI acquired during their current hospital stay (in-house NI) [16]. Hygiene and infection prevention not only reduce individual suffering, but can also make a significant contribution to reducing the length of stay in hospitals and avoiding follow-up interventions. It therefore makes a decisive cross-sectoral contribution to sustainability in the healthcare system.

Nevertheless, infection control can come into conflict with climate and environmental protection, e.g., due to the high energy consumption, the volume of waste, the introduction of ecotoxicologically relevant antimicrobial agents, and the use of disposable products. The consumption of resources required for infection prevention and control (IPC), such as energy, chemicals, materials, water and wastewater treatment, are problematic. This can lead to intrinsic conflicts of interest between optimal infection prevention and optimal climate and environmental protection, which require careful risk assessment. Under no circumstances should patient safety be jeopardized by a reduction in necessary hospital hygiene measures, i.e., the technical expertise secured for infection prevention is the guiding principle. 

Hygiene includes all measures to maintain and improve the health and well-being of individuals and the general public (health science), as well as to prevent and combat infectious diseases and epidemics (health welfare) [17]. Following on from the classic task of hygiene, it is currently necessary for hygienic measures for patient and occupational safety, such as the wearing of disposable medical gloves, to also consider climate and environmental protection aspects in the sense of the One Health or Planetary Health concept [18], [19].

The global market for disposable medical gloves is expected to grow from USD 15.06 billion in 2022 to USD 21.28 billion in 2029, at a compound annual growth rate (CAGR) of 5.1% [20].

According to an environmental statement from a large German hospital (approx. 800 beds), there was an increase in the consumption of disposable medical gloves from 1,058,152 in 2017 [21] to 1,099,996 in 2018 [22] even before the pandemic; in 2020, this hospital consumed 1,370,500 disposable medical gloves [22]. A smaller hospital (approx. 650 beds) in the same hospital group reported consumption of 750,100 units in 2017, 788,500 units in 2018 [23] and 674,300 units in 2019 [24]. For a 200-bed hospital, the consumption of disposable medical gloves was estimated at 750,000 units in 2017 [25]. A study that examined the consumption of personal protective equipment (PPE) at a German university hospital (over 1,000 beds) during the COVID-19 pandemic found a total consumption of 1,558,780 disposable medical gloves in April 2020 [26]. The consumption of disposable medical gloves in outpatient medical facilities has not yet been systematically recorded.

As disposable medical gloves are often used in all areas of medicine and serve for both self-protection (more frequently) and external protection (less frequently) [2], often without a medical indication [27], [28], [29], [30], [31], [32], [33], [34], [35], [36], [37], [38], [39], [40], [41] there is great potential to identify and reduce unnecessary expenditure of resources through the professional assessment of (incorrect) indications. This is also an example of the critical, i.e., exclusively indication-based, use of single-use devices [42].

The use of disposable medical gloves according to indications is associated with at least four advantages:

1. Increases the implementation of hand antisepsis according to indications,
2. improves occupational health and safety (reduction of skin exposure of employees),
3. empathic perception of medical and nursing care through direct hand contact; direct physical contact with the hand instead of the gloved hand, e.g., during personal hygiene by applying cream, during manicures and pedicures, hair washing and body washing (except the anogenital area), conveys caring closeness and social bonding to the patient or person in need of care with a positive influence on the healing process,
4. increases sustainability in the healthcare sector through lower consumption and reduced waste.

This requires a consistent risk assessment/weighting of the areas or activities in which disposable medical gloves are used (see also section 4). In order to maintain the protection goal of patient safety and at the same time not lose sight of the sustainability concept, the assessment of the appropriateness of the use of disposable medical gloves should be applied as a fundamental principle.

With regard to disposable medical gloves, the indication-based use and the associated reduction in consumption has positive effects in several areas. By avoiding non-indicated use, fewer materials are produced, purchased, consumed, discarded, and recycled. This lowers greenhouse gases in the supply chains and reduces environmental pollution caused by chemicals and waste.

In a German study, 90% of the hygiene staff stated that universal use of disposable medical gloves was practiced in the care of severe acute respiratory syndrome coronavirus type 2 (SARS-CoV-2)-positive patients [43]. This universal wearing was even reported for the care of all patients in 30% of all hospitals participating in the study in the period of March/April 2021. It can be assumed that this proportionally non-indicated glove use has increased in the course of the COVID-19 pandemic.

The use of disposable medical gloves affects all medical facilities, many of their employees, and applies to numerous activities. In principle, there is theoretical and empirical evidence that gloves are used not only when there is an indication but also when there is no indication [29]. It can therefore be assumed that the critical indication has the greatest effect. From an infection prevention perspective in particular, it seems effective and sensible to identify and exploit an initial sustainability goal here.

## 3 Definition: disposable medical gloves and distinction from other gloves

The subject of this commentary is **disposable medical gloves** which, since the 2010 revision of the European Union Directive 2007/47/EEC, can be used both as a medical device and as part of PPE for self-protection and protection of others [44]. Disposable medical gloves for these purposes must have defined properties [45] and are subject to the quality criteria of various series of standards, e.g., DIN EN 455-1:2022-04 [46] and ISO 11193-1:2020-08 [47] .

The contents of this commentary refer to disposable medical gloves and not to protective gloves against hazardous chemicals and microorganisms in accordance with DIN EN ISO 374-1:2018-10 [48] or DIN EN ISO 374-5:2017-03 [49] or gloves for non-medical activities (e.g., cleaning).

## 4 Indications and misindications for the use of disposable medical gloves

The non-indicated use of disposable medical gloves can stand in the way of adequate hygiene and infection prevention, e.g., because the required hand antisepsis immediately before aseptic activities appears to be unnecessary. For this reason, the indication will be reviewed below as an example. In addition, section 5 provides information on secondary measures, such as the disinfection of gloves while wearing them, and on the background of various materials.

Disposable medical gloves are used for reasons of patient protection, self-protection, and the combination of both protection goals [50]. Self-protection is the primary reason for their use when a high level of hand contamination with relevant fluids – such as bodily excretions, secretions, excreta or blood – is to be expected (see Table 1 (Tab. 1) and [2], [4], [51]). Self-protection is also the primary indication for patient care if there is reasonable suspicion of the presence or detection of infectious agents with particularly high virulence (see Tab. 1 of the KRINKO recommendation “Infection prevention in the care and treatment of patients with communicable diseases”, updated in 2023 [51]).

Patient protection is the primary reason for using disposable medical gloves for patient contact or contact with the immediate patient environment in the presence of pathogens that are insensitive to alcohol-based hand disinfectants (e.g., Clostridioides difficile) and those with special hygiene requirements, such as multi-resistant (pathogenic) organisms (MDRO). When caring for patients with MDRO, the type of contact (e.g., direct work on the patient if a high level of contamination is expected) should always be the indication for wearing disposable medical gloves; simply entering the room (e.g., to serve food or just to speak to the patient) is not an indication for wearing gloves. The indication is given if a high level of contamination is to be expected, because after massive contamination of the hands, a critical amount for further spread may remain despite hand antisepsis. Studies have shown that 2 to 3 lg colony-forming units (CFU) of methicillin-resistant *Staphylococcus aureus* (MRSA) and *Escherichia coli* remain on the hands after high levels of hand contamination, e.g., after contact with bodily excretions, despite hand antisepsis [52], [53].

Experience and studies have confirmed that incorrect indications for wearing disposable medical gloves arise either from ignorance, an incorrectly perceived indication, a particular need for self-protection or from the group dynamics and/or habits of the medical staff in a facility (“We’ve always done it this way”) [54], [55]. If disposable medical gloves are worn without indication, this not only leads to an additional burden on the climate and resources but must also be viewed critically from an infection prevention perspective [28], [56], [57], [58], [59], [60]. Several studies have shown that hand hygiene compliance is extremely low when wearing disposable medical gloves, especially for the indication with the highest infection prevention relevance for the patient (before aseptic activities, indication 2 of hand hygiene according to WHO [41]). Wearing disposable medical gloves also falsely conveys the idea of impermeability or a false sense of security. In addition, it has been shown that improper removal of disposable medical gloves leads to recontamination of the hands [61], [62]. As the implementation of hand antisepsis is often inadequate even after the gloves have been removed, the transmission of pathogens is therefore less effectively prevented.

**Important note: Table 1** **(Tab. 1)** **lists examples of indications for wearing disposable medical gloves. In accordance with the legal mandate of the KRINKO pursuant to section 23 (1) of the Infection Protection Act (IfSG)** **[63]** **, the indications primarily relate to the prevention of NI in hospitals and other medical or nursing facilities and thus arise for reasons of patient and/or occupational safety. Additional information (e.g., manufacturer's instructions when preparing medication) must also be considered. The specific procedure on site, especially in complex situations, must be agreed upon with the responsible hygiene specialists or occupational medicine and is not the subject of this commentary.**

In addition, it must be considered that for pathogens according to Tab. 1 of the KRINKO recommendation “Infection prevention in the care and treatment of patients with communicable diseases” [51], there may be indications for wearing disposable medical gloves after a risk assessment has been performed (transmission route of the pathogen, possibility of vaccination prevention, specific risk potential for risk groups). One example of this is bed straightening in the presence of scabies.

There is also an indication for occupational safety reasons when handling or administering sensitizing substances, anti-infectives, hormone preparations, and substances with carcinogenic, mutagenic, reprotoxic (CMR) properties (e.g., cytostatics). Among other things, the respective manufacturer’s instructions and special requirements for the reconstitution/preparation of parenterals as well as the available information on the safe handling of medicinal products must be observed [64].

The examples in Table 1 (Tab. 1) of situations in which the use of disposable medical gloves is indicated are contrasted in Table 2 (Tab. 2) with examples in which the use of disposable medical gloves is not indicated but is frequently observed.

### 4.1 Situations in which the wearing of disposable medical gloves is currently recommended from an infection prevention or occupational health and safety perspective, but could be re-evaluated in the future 

An appropriate risk assessment is required before making recommendations on the use of disposable medical gloves. This should be re-evaluated, weighing the appropriateness between patient and occupational safety as well as the concept of sustainability, so that future decisions – even if previous guidelines are confirmed – are made on a new multi-perspective basis. For example, for some activities for which the use of disposable medical gloves has been recommended up to now, the previous requirement could be discussed and either retained or changed after an updated risk assessment. Examples of this could be: the proper performance of blood sampling using safety devices, intravenous injections, or the application of a PVC. A favorable side effect worth mentioning here is the better tactile sense without gloves. The general wearing of gloves when handling medicinal products [64] could also be re-evaluated following a risk assessment [65].

The requirements for wearing disposable medical gloves when handling potentially contaminated waste (medical waste potentially contaminated with blood, secretions, or excretions) are extensively regulated (e.g., in the Technical Rules for Biological Agents 250 (TRBA 250) “Biological agents in health care and welfare facilities” [45]). In most cases, the PPE requirements for waste disposal are very high (e.g., liquid-tight disposable clothing, liquid-tight shoes, etc.) [66], [67]. Here, too, individual current requirements for wearing disposable medical gloves could be re-evaluated, for instance, for the disposal of closed waste bags.

## 5 Further aspects: disinfection of disposable medical gloves and material selection

In addition to avoiding the non-indicated use of disposable medical gloves, there are basically two other options for more sustainable use. In patient care, situations arise in which the use of disposable medical gloves is indicated and there are also a large number of indications (according to the WHO) for hand antisepsis in quick succession. This may be the case in intensive medical care, for example. Wearing disposable medical gloves does not replace the WHO indications for hand antisepsis [3], [37], [41]. Consequently, a very frequent change of disposable medical gloves with hand antisepsis in between would be necessary. This procedure is time-consuming and critical both from the point of view of resource use and infection prevention, as compliance with the above-mentioned requirements is questionable in these situations [42], [68]. A possible co-benefit solution at this point could be disinfection of the gloved hand – considering essential requirements [3], [69]. From the infection prevention perspective, this could increase adherence to the guidelines and possibly also reduce infections [70], [71]. It is also worth mentioning that the effectiveness of disinfection of the disposable medical glove on the gloved hand is higher than on the bare hand [72], [73].

If the use of disposable medical gloves is indicated, the choice of product and/or manufacturer can be additively sustainable. Currently, almost all disposable medical gloves are manufactured in Southeast Asia [74], [75]; there are no regional production sites available that would represent a more advantageous alternative from an economic point of view. While components of conventional nitrile gloves persist in the environment for a long time [76] and are not biodegradable, current developments focus on recycling approaches [77], [78], [79] or on the biodegradability of disposable medical gloves [80], [81], [82]. A broader product range is to be expected and this information could be an additional criterion in a procurement matrix. 

Latex-based disposable gloves can no longer be used as disposable medical gloves due to their high sensitization potential and also offer no advantage in terms of degradability.

## 6 Implementation and options for improving the implementation of the indication-based wearing of disposable medical gloves

To increase the implementation of the indication-based use of disposable medical gloves, it is necessary to communicate this knowledge to the target group and justify the strategy. This should be done iteratively using various training methods and media. The greater challenge, however, appears to be the implementation of the knowledge thus gained into practice, as this requires rethinking of learned, practiced processes. The following multimodal implementation aids can be considered for this purpose:

- Knowledge transfer through education and training programmes,
- training on the relevance of their own actions, role model function of superiors in patient care,
- clear procedural instructions that are easily accessible to every team member,
- adherence/compliance observations by IPC nurses and IPC link nurses,
- psychological and learning theory models for adapting behavior.

Of special importance is the role model function of the IPC link doctors and link nurses for infection control, who are seen as convinced and convincing multipliers in their teams, and at the same time they can assess the subject-specific opportunities and limitations of the measures. Monitoring hand hygiene compliance, the use of disposable medical gloves should also be recorded: standard record sheets or electronic tools can be used and expanded for this purpose [83]. More than 10 years ago, it was shown for several areas that it is possible to reduce the error “gloves instead of hand antisepsis” by optimizing and simplifying processes without additional time or resources [27]. 

The active participation of all those involved, from purchasing to application, as well as patients and their relatives, appears to be fundamentally desirable and helpful in achieving these overarching goals.

All in all, foregoing the use of disposable medical gloves in the absence of an indication can increase sustainability in the healthcare system and at the same time save costs without impairing patient and occupational safety [84], [85], [86]. This requires knowledge transfer, persuasion, behavioral adaptation, and control. A broad social consensus and a common, clearly communicated goal will help to implement this simple measure.

## Notes

This commentary was produced on behalf of the Commission for Hospital Hygiene and Infection Prevention by Prof. Dr. Simone Scheithauer (Head of the working party), Prof. Dr. Heike von Baum, Prof. Dr. Petra Gastmeier, Prof. em. Dr. Axel Kramer and external experts A. Milena Köster, M.A. and Dr. Dieter Müller on a voluntary basis and without influence from commercial groups. From the Robert Koch Institute, Dr. Franziska Lexow was involved. The commentary was prepared by the working party and, after a detailed discussion, agreed by the Commission.

### Competing interests

The author declares to have no competing interests.

## Figures and Tables

**Table 1:**
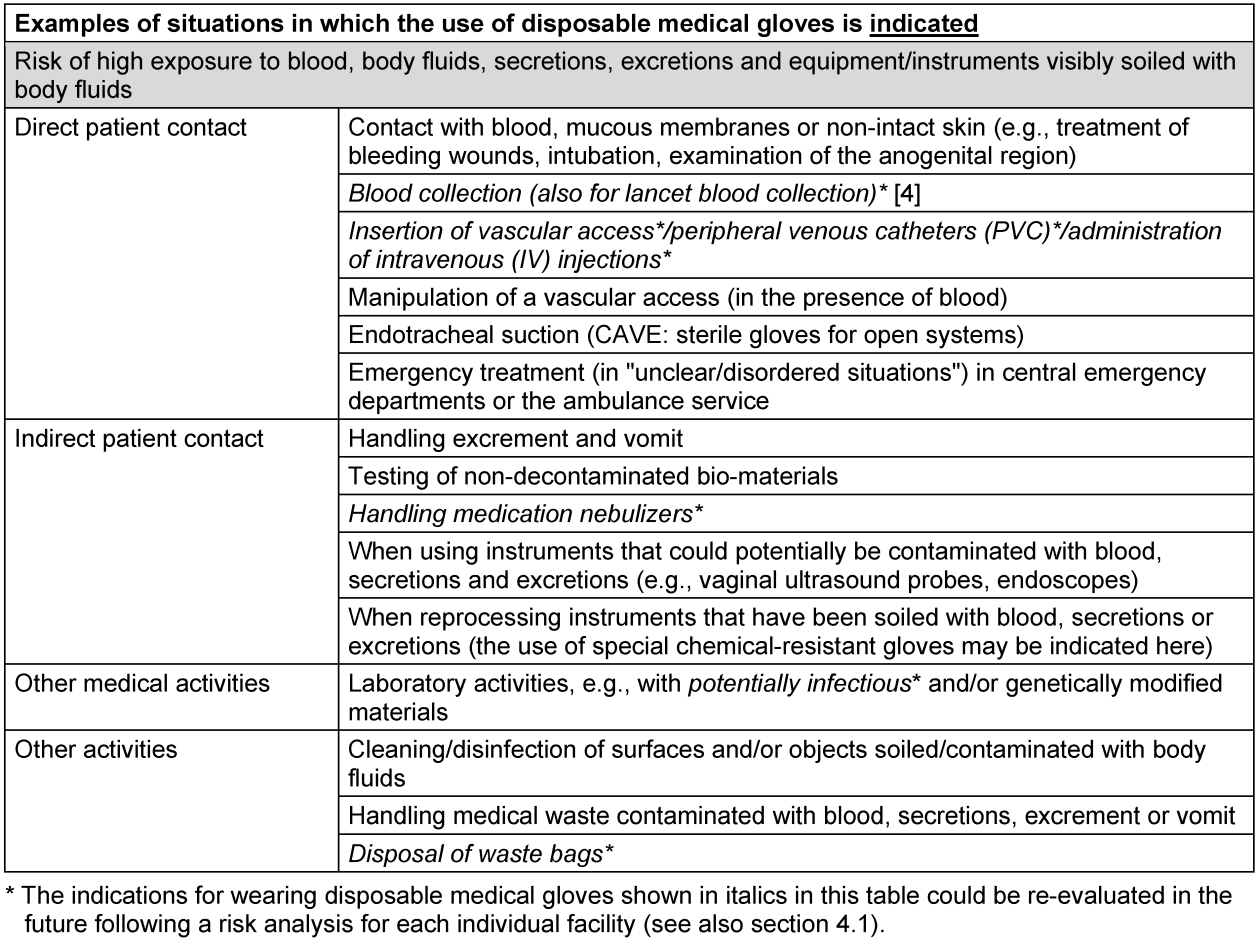
Examples of indications for wearing disposable medical gloves (modified after Bellini et al. [68])

**Table 2:**
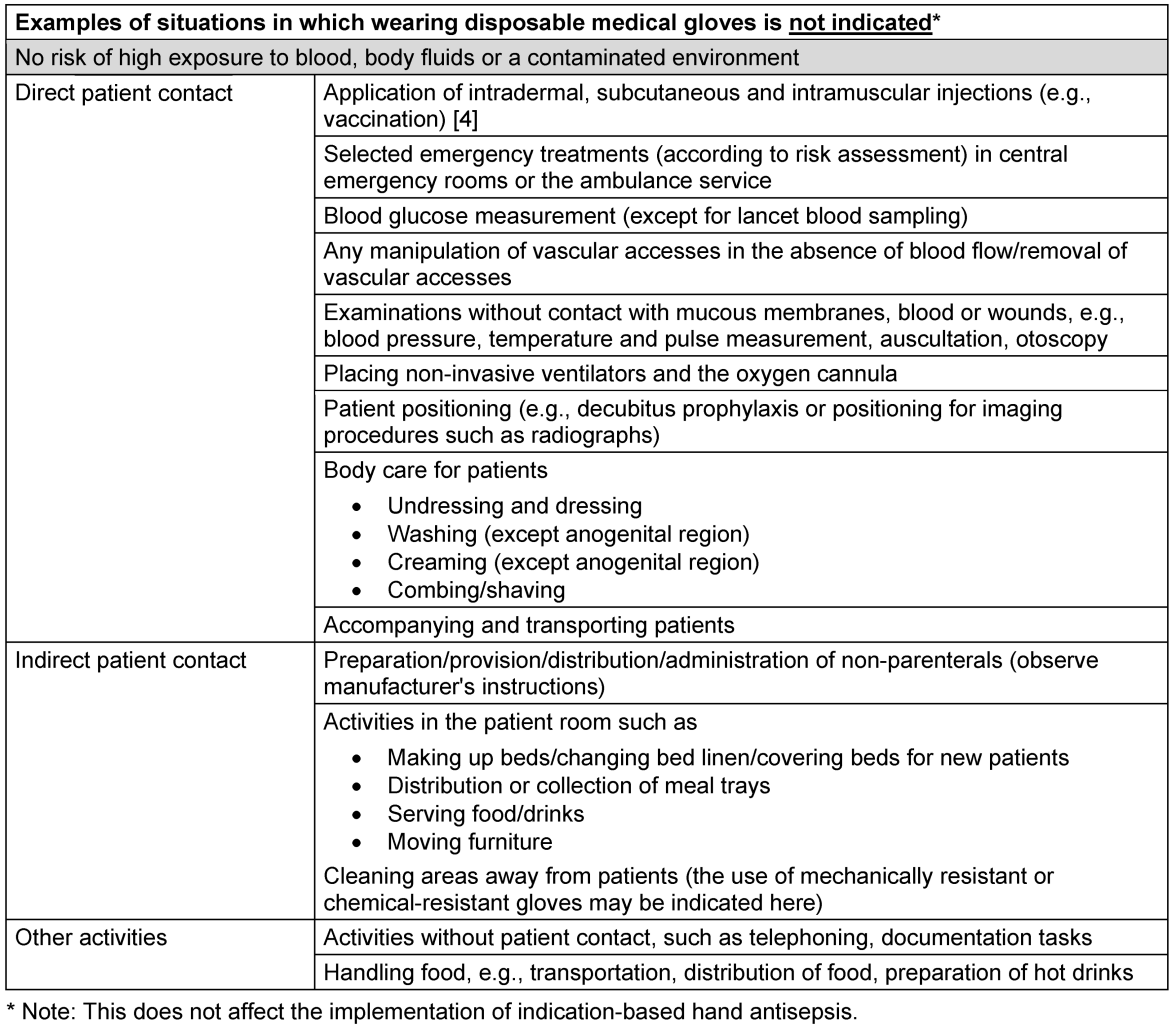
Examples of situations in which wearing disposable medical gloves is not indicated (modified after Bellini et al. [68])
